# Integrated Mechanisms of Anticipation and Rate-of-Change Computations in Cortical Circuits

**DOI:** 10.1371/journal.pcbi.0030082

**Published:** 2007-05-11

**Authors:** Gabriel D Puccini, Maria V Sanchez-Vives, Albert Compte

**Affiliations:** 1 Instituto de Neurociencias de Alicante, Universidad Miguel Hernández-Consejo Superior de Investigaciones Científicas, Sant Joan d'Alacant, Spain; 2 Institut d'Investigacions Biomèdiques August Pi i Sunyer, Barcelona, Spain; L'Unité de Formation et de Recherche Biomédicale de l'Université René Descartes, France

## Abstract

Local neocortical circuits are characterized by stereotypical physiological and structural features that subserve generic computational operations. These basic computations of the cortical microcircuit emerge through the interplay of neuronal connectivity, cellular intrinsic properties, and synaptic plasticity dynamics. How these interacting mechanisms generate specific computational operations in the cortical circuit remains largely unknown. Here, we identify the neurophysiological basis of both the rate of change and anticipation computations on synaptic inputs in a cortical circuit. Through biophysically realistic computer simulations and neuronal recordings, we show that the rate-of-change computation is operated robustly in cortical networks through the combination of two ubiquitous brain mechanisms: short-term synaptic depression and spike-frequency adaptation. We then show how this rate-of-change circuit can be embedded in a convergently connected network to anticipate temporally incoming synaptic inputs, in quantitative agreement with experimental findings on anticipatory responses to moving stimuli in the primary visual cortex. Given the robustness of the mechanism and the widespread nature of the physiological machinery involved, we suggest that rate-of-change computation and temporal anticipation are principal, hard-wired functions of neural information processing in the cortical microcircuit.

## Author Summary

The cerebral cortex is the region of the brain whose intricate connectivity and physiology is thought to subserve most computations required for effective action in mammals. Through biophysically realistic computer simulation and experimental recordings in brain tissue, the authors show how a specific combination of physiological mechanisms often found in neurons of the cortex transforms an input signal into another signal that represents the rate of change of the slower components of the input. This is the first report of a neurobiological implementation of an approximate mathematical derivative in the cortex. Further, such a signal integrates naturally into a neurobiologically simple network that is able to generate a linear prediction of its inputs. Anticipation of information is a primary concern of spatially extended organisms which are subject to neural delays, and it has been demonstrated at various different levels: from the retina to sensori-motor integration. We present here a simple and general mechanism for anticipation that can operate incrementally within local circuits of the cortex, to compensate for time-consuming computations and conduction delays and thus contribute to effective real-time action.

## Introduction

Complex brain functions emerge from the orchestrated activity of neural circuits in the cerebral cortex. This emergent brain activity is dependent on many properties of the cortex, from areal specializations to microcircuit connectivity or cellular and synaptic mechanisms. At the microcicuit level, the cortex is characterized by a fairly stereotyped architecture and physiology [1–5]. These uniform properties of local circuits are likely to operate generic, incremental computations on incoming signals [6]. However, specific instances of the causal relationship between cellular and synaptic mechanisms, network signal processing, and behavioral function have been difficult to identify. This results in part from the myriad experimentally identified physiological mechanisms of synapses and neurons in the cerebral cortex, and their complex reciprocal interactions. Two especially prominent physiological mechanisms are short-term synaptic depression [7] (STD) and intrinsic spike-frequency adaptation [8,9] (SFA). Indeed, synapses between neocortical pyramidal neurons typically show STD [10,11], and most pyramidal neurons in the neocortex display pronounced SFA [8]. Upon sustained stimulation, these well-characterized mechanisms induce, respectively, reductions in synaptic responses or in neuronal firing, and these have been independently related to various neural computations [12–17]. However, the joint effect of SFA and STD on neural information processing has not been scrutinized (but see [18]), despite the fact that they are often associated in cortical circuits and they engage in direct interplay because they act and depend on neuronal firing rate, respectively. Therefore, the combination of presynaptic SFA and STD provides a nontrivial physiologically realistic scenario as yet undefined in terms of neural information processing properties.

We show here, by means of computational modelling and experiments in cortical slices, how the combination of STD and SFA approximates the computation of the rate of change (or derivative) of the input, and how this can be used as the critical building block of a general predictive scheme for neural information processing. Indeed, according to the Taylor approximation for a smooth function, by combining the current stimulus value and its instantaneous rate of change it is possible to estimate the value of the stimulus slightly ahead in time [19,20]. We illustrate this here by proposing a simple, plausible neural architecture that implements a stimulus anticipation response that is in agreement with experimental observations of cortical responses to moving stimuli [21] and can be easily extended to other low-level anticipation or control circuits in the nervous system.

## Results

**Figure 1:**
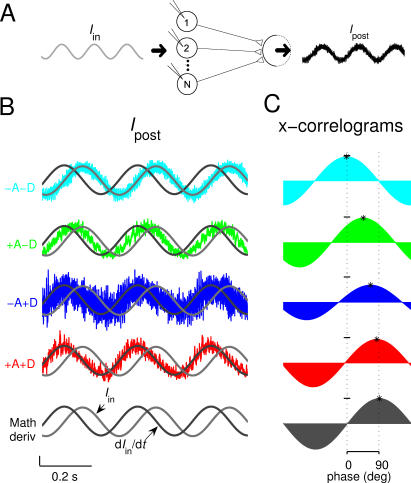
Phase Advances Induced by SFA and STD in the Network Model (A) Scheme of the spiking neuron network employed (*N* = 300). (B) Postsynaptic current *I*_post_ in response to sinusoidal input current *I*_in_ for the networks: no SFA and no STD (−A−D, cyan), SFA only (+A−D, green), STD only (−A+D, blue), and SFA plus STD (+A+D, red). Last panel: mathematical derivative d*I*_in_/d*t*. All panels show *I*_in_ (gray) and d*I*_in_/d*t* (black). Signals are plotted rescaled by their s.d. (vertical scale bar = 1 s.d.). (C) Cross-correlation functions between s.d.-rescaled *I*_in_ and *I*_post_ showing the phase shift induced by the various mechanisms. The off-center location and the height of the central peak are measures of the phase advancement and the signal-to-noise ratio in *I*_post_, respectively.

**Figure 2:**
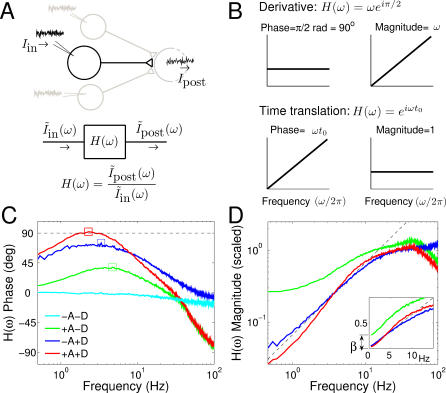
Fourier Analysis of the Network Model Output *I*_post_ upon Stimulation with White Noise Input Current *I*_in_ In a range of low frequencies, networks with STD act as differentiators, and networks with SFA have a low-pass cutoff at neighboring frequencies. (A) Schematic representation of the model and the computation of its transfer function *H*(*ω*) from the Fourier transforms of *I*_in_ 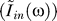 and of *I*_post_ 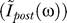 . *H*(*ω*) is an imaginary quantity that is characterized by its magnitude and its phase. (B) Phase and magnitude of the *H*(*ω*) for mathematical derivative (top) or time-shift (bottom) operators. (C) *H*(*ω*) phase and (D) *H*(*ω*) magnitude for Γ = 0.5, τ*_D_* = 400 ms, and τ*_Ca_* = 80 ms in the network models of Figure 1 (same color code). To reduce the variance of *H*(*ω*), a network with *N* = 1,000 presynaptic neurons was used and responses to 50 different white noise realizations were averaged together. Significant phase advancement with linear increasing amplitude occurs for frequencies below 10 Hz when the network includes STD. On a single trial basis, however, only-STD networks produce unsatisfactory, very noisy *I*_post_ (Figures 1 and 4). (D) Every curve is scaled independently to match the initial slopes of |*H*(*ω*)| and to allow for comparisons. Inset: low-frequency range on linear scales.

We built a network model of spiking neurons that integrated an identical injected input current *I_in_* and projected to a postsynaptic neuron through conductance-based synapses (see Materials and Methods). The postsynaptic neuron passively summated incoming postsynaptic currents, and we studied the resulting total current *I_post_* (Figure 1A), as would be obtained in a voltage-clamp experiment. Presynaptic neurons could accommodate their firing through SFA by means of the activation of a calcium-dependent potassium current [9], and their synapses could in addition depress following a phenomenological model calibrated against cortical STD experiments [7]. In the following, we show how this biophysically grounded cortical network model is a robust, physiologically plausible neural implementation of an approximate differentiator operator for low frequencies, so that *I_post_* is a close approximation to the rate of change of the slower components of the input *I_in_*. Our argumentation is based on the two main aspects that characterize the action of the differentiation operator on sinusoidal inputs [*d* sin(*ωt*) / *dt* = *ω* · sin(*ωt* + π/2)]: 90° phase advancement irrespective of sinusoidal input frequency, and modulation amplitude gain proportional to sinusoidal input frequency (Figure 2B).

**Figure 3:**
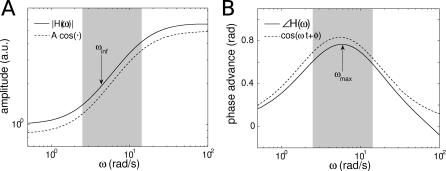
Analytical Calculations Show That the Summation of Asynchronous STD Synapses Produces Derivative-Like Postsynaptic Currents in an Identified Range of Low Frequencies This can be obtained from a white noise input rate (yielding the transfer function *H*(*ω*), solid lines) or from a sinusoidal input rate (dashed lines), through a different set of simplifying assumptions (see Protocol S1). (A) Amplitude of postsynaptic current fluctuations (*x*-axis is Fourier frequency for solid line and frequency of stimulation sinusoid for dashed curve). Amplitude is plotted in arbitrary units, ignoring all dimensional prefactors. *ω*_inf_ denotes the inflection point of the solid curve, around which a straight line is a very good approximation. (B) Phase advancement with respect to input frequency *ω*. *ω*_max_ denotes the frequency at which the maximum phase advancement occurs; around this point the phase is relatively insensitive to frequency *ω*. Dashed regions correspond to the interval [1/τ*_D_*,1/τ*_e_*], in which amplitude grows linearly with *ω* and phase remains approximately constant. Parameters used were τ*_D_* = 0.4 s, Γ = 0.4, *r*_0_ = 20 Hz.

**Figure 4:**
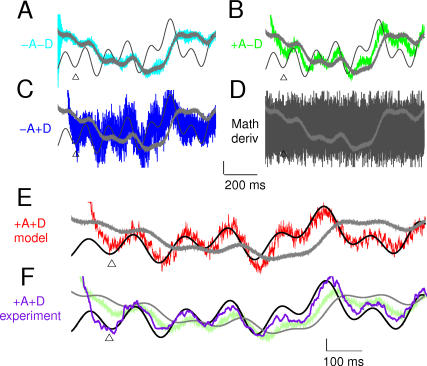
The Network Computed the Rate of Change of an Arbitrary Input, Ignoring High-Frequency Fluctuations (A–E) Same network model as in Figure 1A. All presynaptic model cells receive the same *I*_in_ = *I*_signal_ + *I*_noise_ (gray), with *I*_signal_ the sum of low-frequency sinusoids (see Materials and Methods) and *I*_noise_ a Gaussian white noise. Panels show *I*_post_ for different combinations of SFA and STD. Colors and symbols as in Figure 1. The derivative of *I*_signal_ is plotted (black) to compare with *I*_post_. Unlike the mathematical derivative shown in (D), SFA plus STD (E) acted as a pass-band filter differentiator. (F) Smoothed *I*_post_ for experimentally measured synapses (purple) is also very close to the derivative of *I*_signal_ (all signals equally smoothed for fair comparison). The response of an SFA-only network is superimposed for comparison (pale green). Vertical scale bars = 1 s.d.

### SFA and STD Induce Robust Phase Advancement of a Sinusoidal Input Current *I_in_*

Independently, both STD and SFA are known to advance the phase of a sinusoidal input [12,14]. However, we found that SFA cannot produce sufficient phase advancement to generate the derivative signal, and STD can do so only by strongly reducing the signal-to-noise ratio of *I_post_* (Figure 1B and 1C). Instead, the phase advancement of a sinusoidal *I_in_* was systematically larger and less noisy when both SFA and STD were included in the simulation (case +A+D in Figure 1B), approaching 90° as required by a rate-of-change computation. Adding presynaptic SFA improved the signal-to-noise ratio without canceling other STD effects because synaptic resources were recovered through the decrease of presynaptic firing, so the total postsynaptic charge entry remained approximately constant while synapses gained sensitivity to modulations of the input. This enhanced signal-to-noise and phase-shift effect of the combination of SFA and STD was very robust, as it persisted for significant changes in the parameters that defined the strength and dynamics of both mechanisms in our model (see Figure S1).

### Postsynaptic Response to White Noise Input Reveals Derivative-Like Operation at Low Frequencies

To confirm that the network computed a derivative, in addition to showing that SFA plus STD induced an appropriate phase advancement on sinusoidal signals, we needed to assess that this phase-shift was approximately constant over a significant range of sinusoidal input frequencies, and that the modulation gain in *I_post_* was proportional to the sinusoidal input frequency. We checked this by injecting input currents *I_in_* of broad bandwidth (white noise) into our network model and analyzing the resulting *I_post_*. A spectral analysis of the transfer function 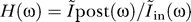 , obtained from the Fourier transforms of *I_in_* and *I_post_* (Figure 2A), allowed for a quantitative assessment of the network's operation on the input signal. Indeed, the complex-valued transfer function *H*(*ω*) of the derivative operator is very different both in phase and magnitude, for instance, from that of a time-shift operator (Figure 2B). In particular, its phase is a constant 90° for all frequencies and its magnitude grows linearly with frequency. When we analyzed the average response of numerous network simulations for the various conditions in our biophysical network, we observed that networks endowed with SFA plus STD and networks with STD but not SFA both generated approximately the characteristics of the derivative for a restricted range of frequencies (Figure 2C and 2D).

### Mathematical Calculations Identify the Range of Validity of Rate-of-Change Computation

We carried out the mathematical analysis of the computational model to explore the parametric conditions for the emergence of derivative-like operation in a depressing, convergent synaptic pathway. As we did numerically above, this can be done in two different ways: in one case we asked about the output to white noise rate fluctuations, and in the other case we computed the output to a sinusoidal rate modulation of given frequency. Both approaches converge approximately to similar solutions (see Figure 3), lead to complementary insights, and revolve around a different set of simplifying assumptions (see Protocol S1 for detailed mathematical derivations). To avoid complications from the firing threshold in presynaptic neurons, we assumed that the size *σ* of temporal modulations in the rate was small compared with the mean presynaptic rate *r*_0_.

Specifically, the transfer function *H*(*ω*) can be obtained exactly under the assumption of asynchronous presynaptic firing [22] and is given by 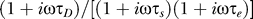 apart from constant prefactors, where ω is the Fourier frequency and 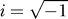 . This shows how the dynamic output of the system depends not only on the two time scales of our synaptic variables, τ*_s_* and τ*_D_* (see Materials and Methods), but also on an emergent time constant τ*_e_* = τ*_D_* /[1 + (1 − Γ) *r*_0_ τ*_D_*]. This time constant is the effective time constant for the relaxation of the synaptic efficacy (*D*) to its steady-state value when stimulated at rate *r*_0_. Because the decay time constant of synaptic conductances τ*_s_* is very small, on the order of a few milliseconds, it does not play a significant role in the dynamics at the low frequencies (<100 Hz) that we are now interested in, and all the dynamics in this frequency range are determined by the time constants τ*_D_* and τ*_e_* that define synaptic depression dynamics. We analyzed the modulus and phase of the transfer function when τ*_s_* → 0 and we found that precisely in the range of frequencies between 1/τ*_D_* and 1/τ*_e_* the modulus of the transfer function has an inflection point at *ω_inf_* and the phase of the transfer function has a maximum at *ω_max_* (see Figure 3). This means that around a given frequency *ω_inf_* in the range [1/τ*_D_*,1/τ*_e_*] 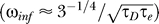 , the modulus of the transfer function is very well approximated by a linear function, and around a given frequency *ω_max_* in the same range 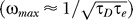 the phase of the transfer function is practically constant. Because neither the modulus is perfectly linear nor the phase perfectly constant in the mathematical sense for a range of frequencies, it is arbitrary to define the range of reasonable approximation by these simpler functions. Thus, considering the range *ω* ∈ [1/τ*_D_*,1/τ*_e_*], it can be shown that the slope of the modulus of the transfer function (plotted versus the frequency *ω*) does not vary by more than a factor of 2 and the phase of the transfer function only drops below half the maximum phase if τ*_e_* < 0.07τ*_D_* (i.e., in the extreme cases when (1 − Γ) τ*_D_ r*_ 0_ > 13). Thus, for frequencies within the interval [1/τ*_D_*,1/τ*_e_*], the modulus of the transfer function is approximately linear and the phase advance sizable. This range of frequencies is large when τ*_e_* is small, i.e., when either *r*_0_ is large or Γ is small. This is in agreement with our numerical exploration of the parametric dependence of approximate differentiation as illustrated in Figure S3A. Therefore, STD in a converging pathway that undergoes significant synaptic summation performs an approximate differentiation, provided the input spectral power is concentrated at frequencies below 1/τ*_e_* and presynaptic firing is modulated around a high firing rate (if we want the range [1/τ*_D_*,1/τ*_e_*] to be one decade long, then *r*_0_ ≥ 30 Hz).

On the other hand, from the mathematical analysis of output responses to a sinusoidally modulated (at frequency *ω*) input rate, one obtains similar results that concur with the transfer function analysis described above (see Figure 3), despite the fact that for the sinusoidal-input case the mathematical derivations require stronger simplifying assumptions (see Protocol S1). However, this analysis gives some additional insight as it reveals at which point in the chain of synaptic events of our model the qualitative properties of the differentiation operator emerge. Indeed, the temporal course of synaptic efficacy (*D*) modulations upon sinusoidally modulated input rate are already phase-shifted (by an amount given by π − arctan *ω*τ*_e_*), but the amplitude of these temporal modulations decreases with input modulation frequency *ω*. It is only after considering summation of responses to a great number of asynchronously firing afferents (mathematically this corresponds to *I*_post_ ≈ *r*(*t*)*D*(*t*), see [22,23]) that the postsynaptic current shows both phase advancement *and* a fluctuation amplitude that increases linearly with *ω*. Thus, the critical mechanisms required for the emergence of a regime of approximate derivative operation at low frequencies are not only STD, but also synaptic summation of asynchronous afferents. However, this is insufficient for differentiation, as high frequency components will be amplified (see Figure 3A) and will dominate the output. The association of presynaptic SFA is what in our circuit filters away high-frequency fluctuations (Figure 2D) to form a robust mechanism of neural differentiation.

### SFA Presynaptic to STD Enhances Signal-to-Noise and Filters High-Frequency Components

When we averaged over many different trials (Figure 2) or analyzed the system mathematically (Figure 3), networks with STD approximated the characteristics of a differentiation operator for low frequencies. However, on a single trial basis only networks with both STD and SFA produced an output with sufficient signal-to-noise ratio to effectively yield the rate of change of arbitrary slowly varying nonsinusoidal currents (Figure 4A–4E). For a given complex-varying input signal, we quantified the similarity of the networks' output with the derivative signal as the magnitude of their cross-correlation's central peak (see Materials and Methods). The SFA plus STD network model yielded a significantly higher cross-correlation (89%) than either of the other networks (SFA only, 74%; STD only, 68%; none, 49%). Notice that our spectral analysis on white noise inputs showed that 90° phase advance (as for a mathematical derivative) applies only for a narrow range of frequencies (0.5–2 Hz, Figure 2C). Figure 4E shows that approximate differentiation is thus not critically dependent on a strict 90° phase shift because in that figure *I_in_* has sinusoidal components with frequencies between 1 and 7.5 Hz. In an additional series of simulations, satisfactory rate-of-change computation (defined as cross-correlation between network output and derivative above 80%) occurred for complex inputs with power content spanning from 1 to 20 Hz (see Figure S2). This range of frequencies corresponds approximately to the range of linear increase of *H*(*ω*) magnitude in Figure 2D. In addition, our white-noise analysis reveals the low-pass filtering properties of networks endowed with SFA, with a cutoff frequency near the upper limit of the frequency range for derivative operation (Figure 2D). Such an effect is advantageous for a system that computes the derivative of low*-*frequency signals, since it leads to the filtering out of high-frequency components, which would otherwise dominate the derivative calculation (Figure 4D). Thus, a network endowed with SFA and STD both ignored high-frequency fluctuations and computed the approximate rate of change of low-frequency input components (≤20 Hz, the range of temporal frequencies coded by neurons in primary visual cortex [24]). This operation was not specific for a particular set of parameters of our model, but it persisted robustly over a significant range of parameter values (see Figure S3).

### Experiments Show Phase Advancement and Magnitude Rescaling of *I_post_* upon Sinusoidal *I_in_,* as Expected for a Derivative

**Figure 5:**
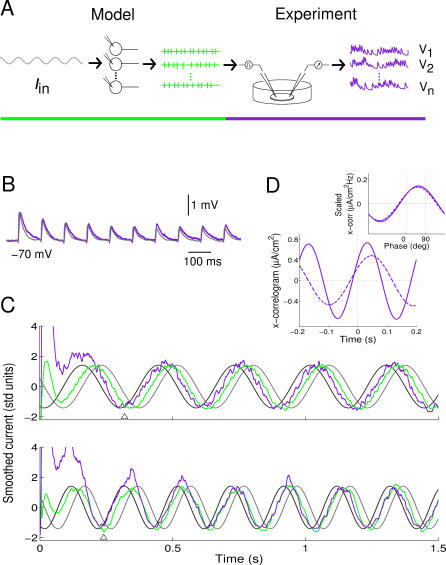
Experimentally, Real Cortical Synapses Produced Constant Phase Advance and Modulation Gain Proportional to Frequency (A) Experiment scheme. Electric shocks were triggered by the sequentially chained spike trains of ∼400 presynaptic model cells. Postsynaptic responses in an intracellularly recorded neuron were analysed off-line, translated in synaptic conductances, and added together to simulate the putative total synaptic conductance evoked by the simultaneous activation of ∼400 presynaptic neurons. The voltage-clamped postsynaptic current *I*_post_ is represented. (B) Control recording for periodic stimulation at 10 Hz (purple) and model trace given by Γ = 0.65 (gray) (Γ is the ratio of peaks of contiguous postsynaptic currents at very high stimulation rates). (C) Smoothed postsynaptic current phase advance in the model with SFA only (green) was enhanced when replacing nondepressing model synapses by real cortical synapses (purple). Results for two different *I*_in_ frequencies are shown (top: 3.5 Hz, bottom: 5 Hz). Signals are plotted normalized to s.d. (computed from triangles onward to avoid transients). (D) Cross-correlation functions between *I*_in_ and non-s.d.–normalized experimental *I*_post_ for the two frequencies (dashed: 3.5 Hz, solid: 5 Hz) overlapped when plotted versus phase and when normalized by stimulation frequency (inset). All data shown here come from the same neuron.

We then wondered whether experimental data in the cortical tissue could support these computational results (Figure 5). We tested experimentally whether our conclusions still held if the schematic model of STD was replaced by real synapses of the cerebral cortex. Presynaptic stimulation was precisely timed to the occurrence of spikes in the adapting presynaptic neurons of the model and delivered by electric shocks to layer 4 of visual cortex slices. The evoked monosynaptic potentials in a connected layer 2/3 neuron were recorded. The convergent architecture of the model was replaced in the experiment by sequential stimulation with the presynaptic spike trains of model neurons and posterior reanalysis to summate those responses as if they had occurred through simultaneous pathways (see Materials and Methods) (Figure 5A). A successful recording required stimulation in stable conditions for a minimum of 30 min. Real synapses advanced the phase of a periodic presynaptic frequency of stimulation in five out of five recordings (phase shift attributable to experimental synaptic plasticity 16.5° ± 8.6°, mean ± s.d., *n* = 5), as it has been theoretically suggested [12]. For two of these recordings, of remarkable stability (>2.5 h, sharp microelectrodes), we stimulated with triggers from presynaptic model neurons excited with two different sinusoidal frequencies (Figure 5C). Both of these recordings concurred with our model results (see Figure 2): for low sinusoidal stimulation frequencies (<10 Hz), phase advancement was practically independent of, and response amplitude gain was approximately proportional to, stimulation frequency (Figure 5D). The fundamental operations of a differentiation operator on low-frequency sinusoidal inputs were therefore accomplished by a neuronal network of intrinsically adapting model neurons projecting convergently through real depressing cortical synapses. We confirmed this by using an arbitrary slowly varying nonsinusoidal current as input for our experimental protocol, which resulted in a reconstructed experimental *I_post_* that approached the rate of change of the input (Figure 4F), in close similarity to the full model's corresponding output (Figure 4E).

### Neurons Can Encode the Temporal Derivative in Their Firing Rate

**Figure 6:**
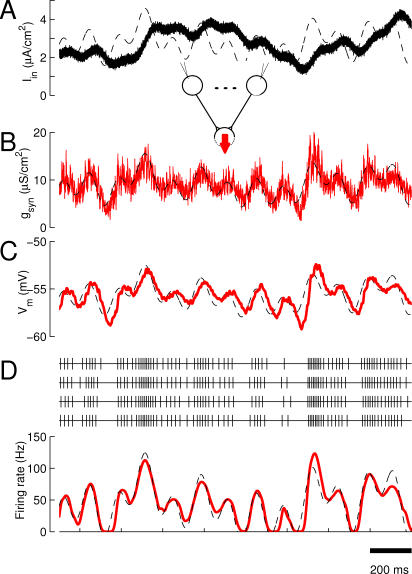
In a Network Scheme with Both SFA and STD, Basic Integration and Spiking Mechanisms in the Postsynaptic Membrane Allow the Postsynaptic Neuron to Encode the Temporal Derivative (A) Current injected into presynaptic neurons (*I*_in_ *,* solid black line). (B) Synaptic conductance opening in the postsynaptic neuron *G_syn_*(*t*). (C) Membrane voltage modulations resulting from synaptic currents and synaptic conductance changes when postsynaptic spiking is inactivated. (D) Sample spike trains (above) and trial-averaged firing rate (below) of the postsynaptic neuron subject to the presynaptic network activity. In all panels, the dashed black curves trace the mathematical derivative of the input, rescaled by the s.d. and recentered by the mean of the plotted signal to allow a direct comparison. The network is exactly as in Figure 4E, with enabled synaptic integration and spiking mechanisms in the postsynaptic neuron. The firing rate curve in (D) was obtained from 400 different simulations and by averaging together normalized bell-curve of s.d. 10 ms centered at the time of spike occurrence. Average postsynaptic firing rate was 64 Hz.

Our combined experimental-modeling approach demonstrated that, for a network endowed with both SFA and STD, voltage-clamped synaptic currents impinging on the postsynaptic neuron *I_post_* follow the temporal derivative of the low-frequency components of the input current to presynaptic cells *I_in_*. Obviously, a number of mechanisms can operate postsynaptically to manipulate and distort this signal (voltage- and activity-dependent channels, etc.). We wondered whether the backbone mechanisms of neural function, synaptic integration and spike generation, posed a problem for the eventual representation of the temporal derivative at the membrane voltage and firing rate levels. We thus repeated the simulations in Figure 4E, only that we now activated synaptic integration (Figure 6C) and both synaptic integration and spike generation (Figure 6D). Our simulations showed that the temporal derivative of *I_in_* could be well-represented not only at the level of synaptic inputs (Figure 6B, cross-correlation peak 89%), but also (and equally well) in the membrane voltage (Figure 6C, cross-correlation peak 92%) and in the firing rate (Figure 6D, cross-correlation peak 95%). Although this shows that the rate-of-change signal can be encoded in spike trains, and propagated to other neurons and networks, an alternative is that this signal is used and manipulated within the postsynaptic neuron before producing a spiking output that is to be propagated downstream. We illustrate this in the following through the construction of an anticipation network.

### Rate-of-Change Network Underlying an Anticipation Neural Scheme in the Cortical Circuit

**Figure 7:**
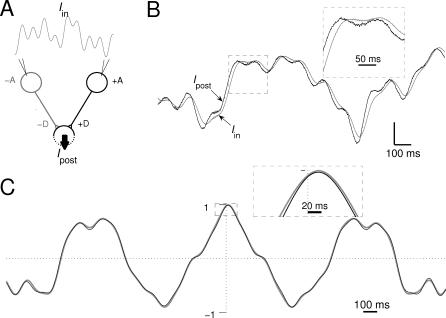
Anticipatory Responses through a Taylor-Approximation–Inspired Neurophysiological Circuit (A) Model scheme for a biological implementation of the first-order Taylor approximation. (B) Anticipatory *I*_post_ response to *I*_in_ = *I*_signal_ of Figure 4 (both currents equally smoothed for readability). Dashed square zoomed in inset. Vertical scale bar represents 0.5 μA/cm^2^. (C) Cross-correlation function between *I*_post_ and *I*_in_ (black) superimposed on autocorrelation function of *I*_in_ displaced by 16 ms (gray). Inset shows the displacement of the central peak (dashed square).

A biological system that has access to the instantaneous rate of change of a signal, can exploit this knowledge in a variety of ways. A straightforward and powerful application is the computation of a prediction of the signal, by adding together the rate of change and the signal itself, as in the first-order Taylor approximation of mathematical calculus [19,20]: *I*(*t* + *τ*) ≈ *I*(*t*) + *τ dI*(*t*) / *dt*. We tested whether the addition in this formula was performed naturally by a postsynaptic neuron receiving inputs both from the network that we have been discussing (contributing *k dI_in_ / dt*) and from a new set of presynaptic neurons that neither adapted nor had depressing synapses (so they contributed *I_in_,* as in Figure 4A). This is represented schematically in Figure 7A and described mathematically by:

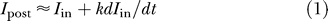
The postsynaptic current *I_post_* was indeed a fixed time interval ahead of the input *I_in_* (Δ*t* ∼ 20 ms. Δ*t* depended on the relative strength of inputs from the two contributing pathways), and predicted it quite accurately (Figure 7B). This can be specifically addressed by computing the cross-correlation function between the output and input currents, and comparing it with an appropriately displaced autocorrelation function of *I_in_* (Figure 7C). The two curves follow each other closely, indicating that indeed *I_post_* is an anticipated version of *I_in_*.

**Figure 8:**
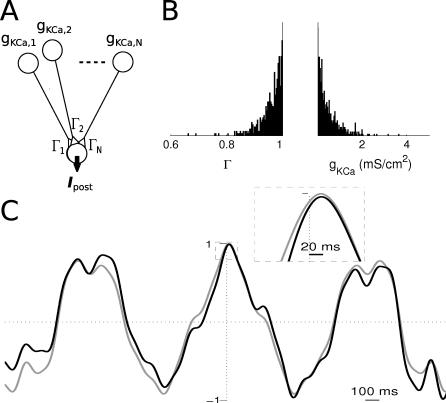
Slightly Degraded Anticipatory Response from a Disordered, Heterogeneous Population of Adapting Neurons and Depressing Synapses (A) Model scheme for a less constrained anticipatory architecture: presynaptic neurons have different values of the *I_KCa_* conductance (*g_KCa_*) responsible for SFA, and their synapses have varying degrees of STD (parameter Γ). (B) Exponential distributions of *g_KCa_* and Γ, by which these parameters are randomly and independently distributed in presynaptic neurons and synapses, respectively. (C) Cross-correlation function between *I*_post_ and *I*_in_ superimposed on autocorrelation function of *I*_in_ displaced by 17 ms. The output is a slightly degraded version of an anticipation of the input. Inset: zoom on central peak (dashed square).

We then wondered how rigidly this anticipatory function was associated with the specific network architecture of Figure 7A. Although it is not an unlikely architecture (two segregated, physiologically distinct pathways could converge from different layers of the cerebral cortex, for instance), the cortical tissue is typically associated with random distribution of properties, rather than with the distinct two-pathway scheme in Figure 7A. Therefore, we tried a network (Figure 8A) where both the strength of SFA in presynaptic neurons and the strength of STD in their synapses were chosen independently at random from exponential distributions (Figure 8B). By choosing an exponential distribution, we ensured that in many cases neurons had little SFA and synapses little STD so the signal suffered little change through the pathway; but a small proportion had sizable SFA and STD, so they contributed a small perturbative additive term, which to leading order resembled a derivative. We thus anticipated such a network to approach the Taylor approximation demonstrated in Figure 7. Indeed, when we injected the same current as in Figure 7 into the heterogeneous population of presynaptic neurons, we obtained a current *I_post_* that followed approximately *I_in_* (see qualitatively similar correlation functions in Figure 8C), advanced by a fixed time window (inset in Figure 8C). Such a randomly heterogeneous network thus also performs an anticipation on the inputs, albeit incorporating further distortions than the network in Figure 7. Therefore, we have identified two biological implementations of a first-order Taylor approximation, through which the cortical network is able to anticipate with various degrees of accuracy the magnitude of its inputs in the immediate future.

### Anticipating Neural Circuits Reproduce the Phenomenology of Motion Extrapolation

**Figure 9:**
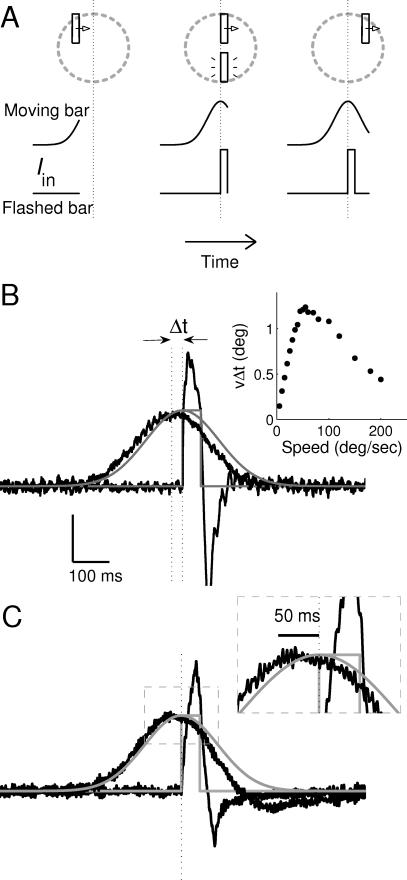
Physiological Implementations of an Anticipatory Network Reproduce Advanced Sensory Responses to Moving Versus Flashed Visual Stimuli (A) Stimulus configurations as in [21]. Incoming current *I*_in_ depended on the position of the moving or flashed bar in the receptive field (top row). For a bar moving with speed *v, I*_in_ was a Gaussian of s.d. *L*/*v*, (*L* = receptive field radius, ∼3° in primary visual cortex). (B) Superposition of the *I*_in_'s for the two stimuli in (A) (gray lines) and corresponding outputs from the model presented in Figure 7 (*I*_post_'s, black traces) show advanced response to moving with respect to flashed stimuli (Δ*t* = 29 ms). Inset: Apparent spatial shift between moving and flashed stimulus from neural responses (*v*Δ*t*) for increasing speed *v*. (C) Same as in (B) but for the model presented in Figure 8. Inset: zoom on the inputs (gray) and responses (black) around the time of the flash onset, showing the advanced response to moving with respect to flashed stimuli. Vertical scale bar in (B) for (B) and (C) represents 0.5 μA/cm^2^.

Our two networks processed slowly varying inputs (as for a stimulus moving into the neurons' receptive field with constant velocity, see Figure 9A) slightly ahead in time as compared with sudden inputs (Figure 9B and 9C, for networks in Figures 7A and 8A, respectively), in agreement with recordings in visual cortex [21]. We computed the spatial mismatch on the visual scene that such differential temporal processing would imply perceptually, to compare with the phenomenology of the “flash-lag effect.” In this perceptual effect, a flashed object appears to lag behind a moving object, even though both are presented in perfect alignment [25,26]. In our model (we show it for the model in Figure 7A, but similar results can be obtained from the model in Figure 8A) and for slow motion (<50 deg/s), the lag distance depended linearly on the velocity of motion of the stimulus (Figure 9B, inset), as observed both neurophysiologically [21] and psychophysically [25]. Our models were also in quantitative agreement with the physiological data [21], but fell short of the visual displacements perceived psychologically [25], suggesting the involvement of additional mechanisms for the perceptual “flash-lag effect.” Also, the model predicted the disappearance of the effect for very rapidly moving stimuli (>100 deg/s, Figure 9B, inset), as has been reported to occur in the retina [27]. Notice that the randomly heterogeneous network of Figure 8A produces an anticipated response that is distorted, especially in its later phase for the moving stimulus (Figure 9C). Thus, for slow motion, our models computed a fixed-interval extrapolation that resulted in shorter latency processing for moving than for flashed stimuli, one of the proposed interpretations of the “flash-lag effect” [25,26,28].

## Discussion

### Physiological Mechanisms for the Rate-of-Change Computation

We have shown that the combination of ubiquitous physiological mechanisms (SFA and STD), often encountered side-by-side in cortical circuits [8,10,11], leads naturally to a network that computes the instantaneous rate of change of its inputs. In isolation STD is more effective than SFA in this respect (see Figure 2), although it has two important drawbacks: it has a very low signal-to-noise ratio, and it does not present a low-pass cutoff at low frequencies to disregard high-frequency synaptic noise. Instead, SFA presynaptic to STD conserves the rate-of-change properties of the STD pathway while solving these two problems. This is achieved through the activity-dependent reduction of the presynaptic rate, and the resulting recovery of depressed synaptic resources. Through this dynamic interplay, the summated excitatory drive into the postsynaptic neuron remains approximately unaffected by SFA while the sensitivity of the synapses to input temporal modulations is significantly enhanced. We thus report novel coordinated interactions between these two mechanisms (see also [18]), which have traditionally been viewed as alternative suppressors of neural excitability. Note that other mechanisms could be invoked to play or enhance some of the roles of SFA in this association. For instance, the spike generation mechanism could provide appropriate low-pass characteristics [29] but it would not control the presynaptic rate to improve the signal-to-noise ratio.

### An Experimental Test for Rate-of-Change Computation in the Cortex

Testing network mechanisms experimentally in vitro is difficult because current experimental techniques do not allow controlled, simultaneous stimulation of large numbers of individual neurons monosynaptically connected to an intracellularly recorded postsynaptic cell. We have circumvented this problem by using one single monosynaptic connection from layer 4 to layer 2/3 of visual cortex, and stimulating it sequentially with the spike patterns of 300–400 presynaptic model neurons. In subsequent off-line analysis we processed recorded voltage responses to eventually add them all together, as if they were arriving simultaneously to the postsynaptic cell. We can thus provide experimental support for STD-induced phase advance and amplitude enhancement (Figure 5), consistent with a derivative operation. Notice, however, that the phase shift observed in our experiment was smaller than would be predicted from our model (Figure 5). We identify a few caveats associated with the experimental approach: on the one hand, inhibition was not blocked in order to avoid epileptiform discharges, particularly in an experiment in which repetitive electrical stimulation was given. Even when we routinely tested the evoked synaptic response at different membrane potentials to ensure that it was strictly excitatory (see Materials and Methods), we cannot rule out that repetitive stimulation did not recruit some inhibitory component. No slow hyperpolarization was ever observed during the protocols, what excludes a significant GABA_B_ activation. Still, we worked with the assumption that if there was some inhibitory shunting, this was constant through each stimulation train. Thus, nonstationary shunting could have been a source of error in our analysis. On the other hand, slow depolarizing NMDA-independent effects were sometimes seen upon repeated electrical stimulation (in three out of five recorded neurons, see example in Figure S4), as has been reported by other authors [30]. These slow changes in membrane potential may reflect the recruitment of other ascending pathways and the action of a neuromodulator, which could be associated with a change in conductance [30]. Theoretically, a change in baseline membrane voltage could also engage postysnaptic voltage-dependent properties, although in our hands the maximum amplitude of the depolarization was very small (1–2 mV). Therefore, our results in Figure 5 are all the more significant, as the predicted effect of STD is qualitatively observed despite the possible presence of these confounding mechanisms, recruited by the nonselectivity of electrical stimulation in the slice.

### Rate-of-Change Computation in Biological Circuits

Here, we explicitly illustrated how derivative calculation can be used in a neural circuit to anticipate incoming inputs, but in fact this basic computation could take part in a variety of information processing operations in neural systems. Indeed, time derivatives are basic computations that are profusely used in complex operations in engineering and physics, typically integrated in control circuits to correct undesired trends in a monitored signal. In biology, implementations of control circuits have already been identified at the biochemical level in molecular biology [31] and at various physiological levels in neuroscience [32,33], suggesting that the computation of the rate of change of a signal is a fundamental operation in biological circuits. A specific neurobiological instance where such a signal has been observed is in sensory receptors of the vestibular system [34], where evidence also supports a neural substrate for the mathematical integration of some Newtonian laws of motion [35,36]. A number of experiments and theoretical studies have also suggested the presence of integrator circuits in the cerebral cortex [37,38]. We provide here the first circuit based on neurophysiological mechanisms of the neocortical microcircuit that is capable of computing the rate of change of electrical current irrespective of high-frequency fluctuations.

### An Anticipation Network Integrated in the Cortical Microcircuit

We have illustrated explicitly how the rate-of-change computation implemented through the interplay between SFA and STD can underlie a physiologically plausible anticipation circuit, mathematically equivalent to a first-order Taylor approximation [19,20]. We suggest that such an anticipatory circuit is integrated in the stereotyped local anatomy and physiology of the neocortex [3,4]. Indeed, specific stereotyped arrangements of connectivity and physiological mechanisms have been observed before [2,3]. The layered architecture of the cerebral cortex provides segregated, highly specific input pathways within a cortical column that could easily embed the anticipatory circuit of Figure 7A [1,4,39]. However, our anticipatory scheme does not even require a very high degree of specificity. Notice that in Equation 1 the term that contributes the rate-of-change signal is comparatively small with respect to the first term (first-order perturbation). The relative magnitude of the two terms (controlled by the relative magnitude of the synaptic strengths in the two pathways) determines both the deviation of the resulting signal from the accurate prediction and the time-window by which the scheme anticipates. Therefore, although the derivative is optimally accomplished by the combination of SFA and STD (Figures 1–5), for the specific case of this anticipation scheme where the rate of change enters only as a small, perturbative term, the sole action of either SFA or STD induces short-time anticipation. This leads us to a formulation of the anticipatory network on much more general physiological terms: a population of neurons highly heterogenous with respect to their degree of SFA and STD produce in a convergently activated postsynaptic neuron a signal that anticipates their own inputs (Figure 8). Thus, cortical networks are endowed with extraordinary flexibility for the implementation of anticipatory circuits: approximate anticipation occurs through the sole action of heterogeneous SFA and STD parameters (Figure 8) or, for higher accuracy, through the specific coordination of these mechanisms (Figure 7).

We propose that our anticipatory schemes are integrated into the cortical local circuit, even though we do not consider mechanisms operating upon postsynaptic neuron function such as postsynaptic SFA or feedback inhibition. This is justified by the fact that we are claiming that the operation occurs between the driving currents to presynaptic (*I_in_*) and postsynaptic (*I_post_*) neurons. Thus, any processing that occurs when postsynaptic neurons are activated would correspond to some further computation on the postsynaptic neuron inputs. In the case of our networks, these inputs *I_post_* would be slightly advanced with respect to inputs to presynaptic cells and thus all ensuing processing will have this anticipation component. Therefore, the networks we identified are interposed conduction elements between two layers of processing, each of which has its own mechanisms to further elaborate information processing. Note, however, that this does not concern feedforward inhibition, which must also be considered a driving element of postsynaptic neurons. The effect of feedforward inhibitory dynamics on these networks thus remains to be elucidated.

### Multilevel Anticipation Circuits in the Cortex

Neurophysiological mechanisms for predictive computations have been proposed before only in the context of learning processes [40–44] or on the basis of the specific functional aspects of circuits with respect to motion processing [21,27]. We present here a plausible hard-wired, bottom-up mechanism that is neither dependent on learning nor specific to a particular stimulus modality or feature. Instead, it performs a lower-level computation: stimuli are predicted based on the empirical fact that inputs typically change continuously and smoothly. It is likely that anticipatory computations are present in the brain at practically all levels of processing, from the very early and low-level aspects discussed here to simple sensory predictions that are based on learned associations, all the way up to the very complex and high-level forward models of sensorimotor control that predict the evolution of the world out of the brain's motor commands [45,46]. Predicting the future state of the surroundings must be such an important tool for behavior that all possible strategies are likely to be exploited, at the corresponding processing level.

### Anticipation as a Basis for the Perception of Predictable Stimuli

We navigate and interact with complex, dynamic environments without typically perceiving any temporal mismatch between our actions and the evolving surroundings. This is a natural capability to expect from a highly evolved organism, but it is also surprising because our sensory neurons transmit delayed signals to the brain [47] and downstream computations incur additional delays [48]. Much delay correction is done by sensorimotor control [45], but delay compensation has also been suggested in sensory systems as an explanation for the “flash-lag effect” [23]. This simple sensory explanation has been challenged by a number of studies that reveal the involvement of mechanisms at a cognitive level [26,49,50]. However, there is physiological evidence of shorter latency neural responses to moving than to flashed stimuli in the visual system [21,27,51], suggesting that sensory anticipation in the visual system is one of the mechanisms of the flash-lag effect. In addition, perceptual studies suggest that faster processing of predictably changing stimuli occurs not only for visual motion, but also across stimulus features and sensory modalities [52,53]. This suggests that one fundamental computation carried out by neural circuits across the cortex is the anticipation of predictable inputs. The mechanistic scheme presented here implements a unifying physiological principle that can underlie low-level anticipation along all stimulus features, sensory modalities, and even beyond sensory cortices, as an integrated delay-correcting module of the cortical microcircuit. This generality together with the ubiquity of the mechanisms invoked and the robustness of the phenomenon in the model and in the experiment, suggests that this anticipatory circuit constitutes a fundamental computational unit of neural information processing in local circuits of the neocortex.

## Materials and Methods

### Biophysical model.

The model consisted of a population of 300–500 presynaptic neurons connected with fast excitatory (AMPA-type) synapses to one postsynaptic neuron. The subthreshold membrane voltage of neurons was modeled according to the equation:

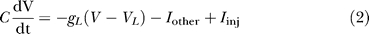
where *C* = 1 μF/cm^2^ is the membrane capacitance, and the leak current is characterized by *V_L_* = −65 mV and *g_L_* = 0.1 mS/cm^2^. *I_inj_* is the injected current, composed by a noisy term 1.5 η(*t*) (where η(*t*) is a Gaussian white noise with unit variance) independent from neuron to neuron and a common input term *I_in_*. In Figure 1 we had *I_in_* = 2.8 + 0.8cos(2*π*3.5*t* + 0.8), while in Figure 4, *I_in_ = I_signal_ + I_noise_*, with *I_signal_* = 2.8 + 0.9cos(2*πt*) + 0.25cos(2*π*2.5*t* + 0.2) + 0.3cos(2*π*3.5*t* + 1.5) + 0.25 · cos(2*π*7.5*t* + 1.8) and *I_noise_* = 1.5 η(*t*). In Figure 7B we used *I_in_ = I_signal_*, the same *I_signal_* as in Figure 4.

Presynaptic neurons fired an action potential whenever their membrane potential reached the threshold *V_th_* = −60 mV, at which point the membrane potential was reset to *V_reset_* = −70 mV (integrate-and-fire model). The potential was then held fixed at *V_reset_* for a refractory period of 2 ms. Other currents were incorporated at the location of *I_other_:* presynaptic adapting cells could have a calcium-dependent potassium current *I_other_ = I_KCa_* for the generation of SFA, and postsynaptic neurons could have a synaptic input current *I_other_ = I_post_* due to the activity of their presynaptic partners.

Following [9], we modelled the calcium-dependent potassium current *I_KCa_* as:

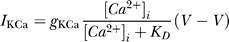
with *K_D_* = 30 mM, *V_K_* = −80 mV, and *g_KCa_* = 5 mS/cm^2^ (except in Figure 8 and Figure S1, as indicated). The dynamics of intracellular calcium concentration [*Ca* ^2+^]*_i_* was modeled as follows: at each action potential, [*Ca* ^2+^]*_i_* increased instantaneously by an additive constant α*_Ca_* (with α*_Ca_* = 0.2 μM/spike) due to the influx of calcium, and then decreased exponentially with τ*_Ca_* = 80 ms (except for Figures S1 and 2, as indicated).

Voltage-clamped excitatory synaptic currents impinging on the postsynaptic neuron were modeled as *I_post_* = *G_syn_*(*t*)(*V_hold_* − *V_syn_*) where *V_hold_* = −65 mV, *V_syn_* = 0, and *G_syn_*(*t*) denotes the total synaptic conductance open in the postsynaptic membrane. *G_syn_*(*t*) decayed exponentially to zero with a time constant *τ_s_* = 2 ms, and when a presynaptic spike occurred, *G_syn_* rose instantaneously to *G_syn_ + g_syn_ D,* where *g_syn_* represents the maximal synaptic conductance in a unitary synaptic connection (*g_syn_* = 0.24 mS/cm^2^ except for Figure 6 where *g_syn_* = 0.7 mS/cm^2^, and except for depressing synapses in Figures 7, 8, 9B, and 9C, which have *g_syn_* = 1.2 mS/cm^2^, *g_syn_* = 0.75 mS/cm^2^, *g_syn_* = 2.85 mS/cm^2^, and *g_syn_* = 3.6 mS/cm^2^, respectively), and *D* is the depression variable of the corresponding presynaptic neuron (modelled as in [7]). For nondepressing synapses, *D* = 1. In depressing synapses, each time a spike arrived at a presynaptic terminal, *D* was reduced multiplicatively to Γ*D*, with 0 ≤ Γ ≤ 1 (we took Γ = 0.65, except for supporting figures, as indicated). The recovery of the synaptic resources toward the maximal value (*D* = 1) occurred between presynaptic action potentials: *τ_D_dD* / *dt* = 1 − *D*, with τ*_D_* = 0.4 s (except for Figures S1 and S3, as indicated). In Figure 6 postsynaptic responses at the level of membrane voltage and firing rate were obtained using an integrate-and-fire postsynaptic neuron model (Equation (2) with *I_other_* = *G_syn_*(*t*) (*V* − *V_syn_*) and corresponding descriptions above).

### Experiments.

Slices of the visual cortex of adult (3–6 mo) ferrets were prepared as described previously [54]. Experiments were approved by the local ethical committee and done in accordance with Spanish regulatory laws (BOE 256; 25/10/1990) that comply with European Union guidelines on protection of vertebrates used for experimentation (Strasbourg 3/18/1986). After preparation, slices were placed in an interface-style recording chamber (Fine Science Tools, http://www.finescience.com) and bathed in ACSF containing (in mM): NaCl, 124; KCl, 2.5; MgSO_4_, 2; NaHPO_4_, 1.25; CaCl_2_, 2; NaHCO_3_, 26; and dextrose, 10, and was aerated with 95% O_2_, 5% CO_2_ to a final pH of 7.4. Bath temperature was maintained at 34–35 °C. Intracellular recordings were initiated after 2 h of recovery.

Sharp intracellular recording electrodes were formed on a Sutter Instruments (http://www.sutter.com) P-97 micropipette puller from medium-walled glass and bevelled to final resistances of 50–100 MΩ. Sharp electrodes were considered adequate given the long duration (>2 h) of the protocols run consecutively in each recorded neuron. Micropipettes were filled with 2 M KAc. Recordings were digitized and acquired using a data acquisition interface and software from Cambridge Electronic Design (http://www.ced.co.uk). For the study of synaptic depression, electrical stimulation (0.1 ms, 10–300 μA) was delivered by means of a WPI A-360 stimulus isolation unit (http://www.wpiinc.com) that prevents electrode polarization. A concentric bipolar stimulating electrode (FHC, http://www.fh-co.com) was placed in layer 4 and the postsynaptic neurons recorded in layer 2/3. Only monosynaptic connections were included, the criteria being: reliably evoked synaptic potentials (no failures) of constant amplitude and shape and with a constant latency (jitter <1 ms) of 1.5–3 ms [55]. The evoked synaptic potentials (amplitudes 3–5 mV) were routinely examined at different membrane potentials to ensure that they only contained an excitatory component. During presynaptic electrical stimulation, neurons were hyperpolarized to 80 mV ± 2 mV to prevent action potential firing. To simulate in vitro the convergent input of a large number of presynaptic cells (*n* = 300–400) on to a postsynaptic neuron, we applied the stimulation trains generated by each model presynaptic neuron (duration 1–1.5 s) sequentially (5-s recovery periods). The bridge-balance was monitored and adjusted in between trains. The putative postsynaptic response was reconstructed off-line as if those trains of stimuli had been simultaneous (see below). All synaptic and injection currents in Figures 4F and 5 were smoothed (20-ms square kernel) for readability. Trains of ten electric shocks at 10 Hz were delivered before and interspersed during the recording to verify both the presence of depressing synapses and the relative invariability of depression with respect to previous stimulations (Figure 5B).

### Off-line analysis.

We analyzed off-line the intracellularly recorded membrane potential during each series of extracellular stimulation trains, measuring the size of excitatory postsynaptic potentials at the time of each stimulus trigger *t_spk_* (difference between maximum in a 4-ms window following stimulation artifact and voltage value right before the artifact). A reconstructed membrane potential was then generated by multiplying the measured size of each excitatory postsynaptic potential by the difference of exponentials:

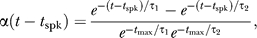
with *t* > *t_spk_* and *t_max_* = *τ*_1_ *τ*_2_ log(*τ*_1_ / *τ*_2_) / (*τ*_1_ − *τ*_2_), where *τ*_1_ = 10 ms and *τ*_2_ = 2 ms. By doing this, we reconstructed a smoother voltage time series that only incorporated events associated with our stimulation protocol. We used this approach to remove all spontaneous synaptic activity not associated with stimulation events, which could otherwise have interfered with our analysis. The resultant reconstructed potential was then cut in individual responses *V_i_,* and translated into putative synaptic conductance changes from the equation *G_i_*(*t*) = −*I_i_*(t) / *V_i_*(*t*) with *I_i_* = *C dV_i_* / *dt* + *g_L_*(*V_i_* – *V_L_*) – *I*. The values of *C* and *g_L_* were derived from voltage responses to hyperpolarizing pulses of current injected intracellularly (for the cell in Figure 5 *C* = 0.31 nF and *g_L_* = 0.02 μS). The resulting synaptic conductance traces were added together, replicating the postsynaptically integrated conductance evoked by ∼300 simultaneously active presynaptic neurons: *G_syn_*(*t*) = ∑*G_i_*(*t*). Total synaptic conductance was translated to voltage-clamped postsynaptic current by means of *I_post_* = −*G_syn_ V_hold_*. Thus, the experimental information incorporated into our analysis was that concerning the synaptic efficacy change operated by in vitro synaptic plasticity.

### Cross-correlation.

The cross-correlation function between the input current *I_in_* and the postsynaptic current *I_post_* (see Figures 1, 5, and S1) was calculated by applying the cross-correlation theorem, i.e., anti-transforming the product of the Fourier transform of one function by the complex conjugate of the other function. The mean and standard deviation (s.d.) of each signal were computed starting from 0.2 s in Figure 1 and from the first minimum following the transient of the signal (indicated by upward-pointing triangles) in Figures 4 and 5. Two measures were extracted from each cross-correlation function: the amplitude and the phase of the central peak (see inset in Figure S1A). The amplitude of the cross-correlation's central peak is a measure of the signal-to-noise ratio of *I_post_*, and it has a maximal value of 1 (we denote this as 100% in Figure S1) when the cross-correlation is computed on normalized values for the two signals (*I_post_* and *I_in_*). To maintain information about the magnitude of current modulations in *I_post_*, we did not normalize by its s.d. when computing the cross-correlation functions in Figure 5D. On the other hand, the phase of the cross-correlation's central peak gives a measure of the phase displacement of *I_post_* relative to *I_in_*. These measures provide a way to determine whether *I_post_* approximates the derivative of a sinusoidal *I_in_:* the maximum of the cross-correlation should be close to 100% and the phase of the central peak close to 90° (upper right corner in Figure S1A). These criteria were central to our exploration of how robust the model was to parameter change in the mechanisms of SFA and STD (Figure S1). Similarly, we use the cross-correlation maximum as a measure of the approximation to the derivative in the case of more complex *I_in_* (Figures 4, 6, and S2).

## Supporting Information

Figure S1 Robustness of Network-Induced Phase-Shift of Sinusoidal Currents as Parameters for STD (Γ, τ*_D_*) and for SFA (τ*_Ca_*, *g_KCa_*) Are Varied (102 KB TIF)

Figure S2 Output of a Network with SFA and STD Performs a Rate-of-Change Computation (up to a Correlation of 80%) for Inputs with Power Content Concentrated below 20 Hz (260 KB TIF)

Figure S3 Results of the Computational Model in Figures 2 and 4 Are Robust to Parameter Modifications (353 KB TIF)

Figure S4 Sample Raw Data from the Experiment in Figure 5
(189 KB TIF)

Protocol S1 Explicit Mathematical Derivations Described in the Text (91 KB PDF)

## Abbreviations

s.d.: standard deviation
SFA: spike-frequency adaptation
STD: short-term synaptic depression
