# Supplementary material for: Integrated Mechanisms of Anticipation and Rate-of-Change Computations in Cortical Circuits
Source: PLoS Comput Biol. 2007 May 11;3(5):e82. doi: 10.1371/journal.pcbi.0030082 (PMC1866356; doi:10.1371/journal.pcbi.0030082)
Supplement: Supplementary file 1 [file pcbi.0030082.sd001.pdf]

Protocol S1

We carried out mathematical analysis of the feedforward network endowed with STD in its synapses in order to understand the parametric robustness of the results in Figure 2. Following the network simulations in the main text, we analyzed mathematically two different situations: presynaptic neuron firing rate is sinusoidally modulated, and presynaptic neuron firing is very irregular and can be modeled as a Gaussian white noise. For yet another mathematical approach to this issue see [55].

Analytic transfer function We analyzed the case when the train of spikes arriving at a typical synapse is very variable and can be modeled by a fluctuating rate $r(t) = r_0 + \sigma\eta(t)$, where r_0 is the average rate of incoming spikes, σ is the standard deviation of fluctuations in the rate and $\eta(t)$ is a Gaussian white noise of unit variance. When presynaptic neurons fire asynchronously with rate $r(t)$, the synaptic dynamics defined by (see Materials and Methods)

$$\begin{aligned}\frac{dD}{dt} &= \frac{1-D}{\tau_D} - (1-\Gamma)D\sum_i \delta(t-t_i)\\ \frac{dG_{\rm syn}}{dt} &= -\frac{G_{\rm syn}}{\tau_s} + g_{\rm syn}D\sum_i \delta(t-t_i)\end{aligned}$$

($\{t_i\}$ are the times of presynaptic spikes and $\delta(t-t_i)$ is the impulse (delta) function centered at the time of spike occurrence) can be written in a mean-field formulation as (see [54])

$$\frac{dD}{dt} = \frac{1-D(t)}{\tau_D} - (1-\Gamma)D(t)[r_0+\sigma\eta(t)] \quad (1)$$

$$\frac{dG_{\rm syn}}{dt} = -\frac{G_{\rm syn}(t)}{\tau_s} + g_{\rm syn}D(t)[r_0+\sigma\eta(t)] \quad (2)$$

In voltage clamp, $G_{\rm syn}(t)$ is proportional to the postsynaptic current I_{post} so we wanted to derive the transfer function from the input rate to $G_{\rm syn}(t)$. Notice that Eq. (1) can be rewritten in terms of the effective time constant of synaptic resource depletion $\tau_e = \tau_D/(1+(1-\Gamma)r_0\tau_D)$ and the steady-state synaptic efficacy $D_\infty = \tau_e/\tau_D$ as

$$\frac{dD}{dt} = \frac{D_\infty - D(t)}{\tau_e} - (1-\Gamma)D(t)\sigma\eta(t) \quad (3)$$

We computed the Fourier transform of Eqs. (3) and (2) to obtain

$$\begin{aligned}i\omega\tilde{D} &= \frac{D_\infty\delta(\omega)-\tilde{D}}{\tau_e} - (1-\Gamma)\sigma\int_{-\infty}^{\infty} d\omega'\tilde{D}(\omega')\tilde{\eta}(\omega-\omega')\\ i\omega\tilde{G}_{\rm syn} &= -\frac{\tilde{G}_{\rm syn}}{\tau_s} + g_{\rm syn}\tilde{D}r_0 + g_{\rm syn}\sigma\int_{-\infty}^{\infty} d\omega'\tilde{D}(\omega')\tilde{\eta}(\omega-\omega')\end{aligned}$$

These equations can be rewritten as:

$$\tilde{D} = \frac{D_\infty \delta(\omega)}{1+i\omega\tau_e} - \frac{(1-\Gamma)\sigma\tau_e}{1+i\omega\tau_e}\int_{-\infty}^{\infty} d\omega' \tilde{D}(\omega')\tilde{\eta}(\omega-\omega') \quad (4)$$

$$\tilde{G}_{\rm syn} = \frac{g_{\rm syn}\tau_s}{1+i\omega\tau_s}\left[r_0 D_\infty \delta(\omega) + \sigma\left(1-\frac{(1-\Gamma)\tau_e r_0}{1+i\omega\tau_e}\right)\times\cdots \right.$$
$$\left. \int_{-\infty}^{\infty} d\omega' \tilde{D}(\omega')\tilde{\eta}(\omega-\omega')\right] \quad (5)$$

where in (5) we made use of (4) once. We now used equation (4) again into the integrand of (5) and obtained:

$$\tilde{G}_{\rm syn} = \frac{g_{\rm syn}\tau_s}{1+i\omega\tau_s}\left[r_0 D_\infty \delta(\omega) + \sigma\left(1-\frac{(1-\Gamma)\tau_e r_0}{1+i\omega\tau_e}\right)\left(D_\infty\tilde{\eta}(\omega) - \cdots \right.\right.$$
$$\left.\left. \int_{-\infty}^{\infty} d\omega' \frac{(1-\Gamma)\tau_e\sigma\tilde{\eta}(\omega-\omega')}{1+i\omega'\tau_e}\int_{-\infty}^{\infty} d\omega'' \tilde{D}(\omega'')\tilde{\eta}(\omega'-\omega'')\right)\right] \quad (6)$$

We now sought an expression for $\langle \tilde{G}_{\rm syn}\tilde{\eta}^*\rangle$, where $\langle\cdot\rangle$ indicates average over many different white noise realizations $\eta(t)$. This can be done directly on equation (6), taking into account that for a white noise process $\eta(t)$ the lowest order non-vanishing moment are second-order correlations $\langle\tilde{\eta}\tilde{\eta}^*\rangle$, and we neglected all higher order moments.

$$\langle \tilde{G}_{\rm syn}\tilde{\eta}^*\rangle = \frac{g_{\rm syn}\tau_s\sigma D_\infty}{1+i\omega\tau_s}\left(1-\frac{(1-\Gamma)\tau_e r_0}{1+i\omega\tau_e}\right)\langle\tilde{\eta}\tilde{\eta}^*\rangle \quad (7)$$

This yields immediately the transfer function for this synapse at all frequencies, as a function of the parameters of the synapse (τ_s, $g_{\rm syn}$, τ_D, Γ) and the mean r_0 and variance σ^2 of presynaptic afferent firing:

$$H(\omega) = g_{\rm syn}\tau_s\sigma\left(\frac{\tau_e}{\tau_D}\right)^2 \frac{1+i\omega\tau_D}{(1+i\omega\tau_s)(1+i\omega\tau_e)} \quad (8)$$

where $\tau_e = \frac{\tau_D}{1+(1-\Gamma)r_0\tau_D}$.

The module and phase of the transfer function are now easily derivable and read:

$$|H(\omega)| = g_{\rm syn}\tau_s\sigma\left(\frac{\tau_e}{\tau_D}\right)^2\sqrt{\frac{1+\omega^2\tau_D^2}{(1+\omega^2\tau_s^2)(1+\omega^2\tau_e^2)}} \quad (9)$$

$$\angle H(\omega) = \arctan\frac{\omega(\tau_D-\tau_s-\tau_e-\omega^2\tau_D\tau_s\tau_e)}{1+\omega^2(\tau_D\tau_s+\tau_D\tau_e-\tau_s\tau_e)} \quad (10)$$

Because $\tau_s \ll \tau_D, \tau_e$, Eqs. (9) and (10) can be approximated by

$$|H(\omega)| \simeq g_{\rm syn}\tau_s\sigma\left(\frac{\tau_e}{\tau_D}\right)^2\sqrt{\frac{1+\omega^2\tau_D^2}{1+\omega^2\tau_e^2}} \quad (11)$$

$$\angle H(\omega) \simeq \arctan\frac{\omega(\tau_D-\tau_e)}{1+\omega^2\tau_D\tau_e} \quad (12)$$

From these easier expressions we extracted the inflection point of $|H(\omega)|$ and the maximum of $\angle H(\omega)$ analytically. These were found to occur at the points

$$0 = \left.\frac{d^2|H(\omega)|}{d\omega^2}\right|_{\omega_{inf}} \quad \Rightarrow \quad \omega_{inf} \simeq \frac{1}{\sqrt{\tau_D \tau_e}} \frac{1}{\sqrt[4]{3}} \sqrt{\sqrt{1+\left(\frac{\tau_e}{\sqrt{3}\tau_D}\right)^2} - \frac{\tau_e}{\sqrt{3}\tau_D}} \tag{13}$$

$$0 = \left.\frac{d\angle H(\omega)}{d\omega}\right|_{\omega_{max}} \quad \Rightarrow \quad \omega_{max} \simeq \frac{1}{\sqrt{\tau_D \tau_e}} \tag{14}$$

Both the inflection point of $|H(\omega)|$ and the maximum of phase shift $\angle H(\omega)$ occur at frequencies between $1/\tau_D$ and $1/\tau_e$. This shows that in this range of frequencies the transfer function module is approximately linear with frequency (the higher order correction to the linear approximation is already third order) and the phase of the transfer function is maximal and approximately constant (the linear approximation to the phase has zero slope). It is therefore in this frequency range that synaptic depression is acting as an approximate differential operator. However, this operation will be masked by higher frequency oscillations, which have a larger $|H(\omega)|$, unless the input is low-pass filtered appropriately. This is what SFA accomplishes in our simulations.

Response to sinusoidal input When the rate of spikes arriving at a typical synapse is sinusoidally modulated at a frequency ω around a mean rate r_0, the equations for the synapse read

$$\frac{dD}{dt} = \frac{1-D(t)}{\tau_D} - (1-\Gamma)D(t)(r_0 + \sigma\cos\omega t) \tag{15}$$

$$\frac{dG_{\text{syn}}}{dt} = -\frac{G_{\text{syn}}(t)}{\tau_s} + g_{\text{syn}}D(t)(r_0 + \sigma\cos\omega t) \tag{16}$$

Again, Eq. (15) can be rewritten in terms of the effective time constant of synaptic resource depletion $\tau_e = \tau_D/(1+(1-\Gamma)r_0\tau_D)$ and the steady-state synaptic efficacy $D_\infty = \tau_e/\tau_D$ as

$$\frac{dD}{dt} = \frac{D_\infty - D(t)}{\tau_e} - (1-\Gamma)D(t)\sigma\cos\omega t \tag{17}$$

Equation (17) is a first-order linear ordinary differential equation, whose solution $D(t)$ can be formally written as:

$$D(t) = D_0 e^{-t/\tau_e - (1-\Gamma)\sigma\sin(\omega t)/\omega} + \cdots + \frac{D_\infty}{\tau_e}\int_0^t e^{-t'/\tau_e} e^{-\frac{(1-\Gamma)\sigma}{\omega}[\sin\omega t - \sin\omega(t-t')]} dt' \tag{18}$$

To obtain a more tractable functional expression for $D(t)$ from Eq. (18), we used the approximation.

$$e^{-\frac{(1-\Gamma)\sigma}{\omega}[\sin\omega t - \sin\omega(t-t')]} \simeq 1 - \frac{(1-\Gamma)\sigma}{\omega}\left[\sin\omega t - \sin\omega(t-t')\right] \tag{19}$$

This allowed for the explicit calculation of the integral in Eq. (18), yielding

$$
\begin{aligned}
D(t) &\simeq D_0 e^{-t/\tau_e-(1-\Gamma)\sigma\sin(\omega t)/\omega} + \cdots \\
&+D_\infty e^{-t/\tau_e}\left[1-(1-\Gamma)\sigma\tau_e\left(\frac{\sin\omega t}{\omega\tau_e}-\frac{1}{1+\omega^2\tau_e^2}\right)\right]\cdots \\
&+D_\infty\left[1-\frac{\sigma}{r_0}\frac{\tau_D}{\tau_D+\tau_e}\frac{\cos(\omega t-\varphi)}{\sqrt{1+\omega^2\tau_e^2}}\right] \qquad (20)
\end{aligned}
$$

where we used trigonometric equalities and we defined $\varphi = \arctan(\omega\tau_e)$.

The first two terms in (20) reflect transients in the response, so we focused on the third term, the steady-state solution. We see that, in the steady state, $D(t)$ has a phase offset with respect to the input rate oscillations of $\pi - \varphi$ and has an amplitude of the oscillations that decreases with oscillation frequency ω. This does not correspond to a derivative operation, which requires an amplitude that grows linearly with frequency.

We then looked at how the synaptic response $G_{\rm syn}(t)$ reacted to these synaptic efficacy modulations $D(t)$, using Eq. (16). However, because τ_s is very small with respect to all other time constants in the system and with respect to the range of stimulation frequencies ω of our interest, we decided to simplify the analysis by equating the left-hand-side of (16) to zero. We then obtained

$$
\begin{aligned}
G_{\rm syn}(t) &\simeq g_{\rm syn}\tau_s D_\infty\left(r_0+\sigma\cos\omega t\right)\left[1-\frac{\sigma}{r_0}\frac{\tau_D}{\tau_D+\tau_e}\frac{\cos(\omega t-\varphi)}{\sqrt{1+\omega^2\tau_e^2}}\right] = \\
&= g_{\rm syn}\tau_s D_\infty r_0\left\{1-\frac{1}{2}\frac{\sigma^2}{r_0^2}\frac{\tau_D}{\tau_D+\tau_e}\frac{\cos\varphi+\cos(2\omega t-\varphi)}{\sqrt{1+\omega^2\tau_e^2}}+\cdots\right. \\
&\left.\cdots+\frac{\sigma}{r_0}\frac{\tau_e}{\tau_D}\frac{\tau_D}{\tau_D+\tau_e}\sqrt{\frac{1+\omega^2(\tau_D^2+\tau_e^2)}{1+\omega^2\tau_e^2}}\cos(\omega t+\phi)\right\} \qquad (21)
\end{aligned}
$$

where we defined the phase $\phi = \arctan\left\{\omega\tau_D/\left[1+\omega^2\tau_e(\tau_e+\tau_D)\right]\right\}$

So, the leading order temporal modulation (in terms of σ/r_0) is the third term in (21), and it is a sinusoidal modulation with amplitude and phase given by

$$
\begin{aligned}
\text{amplitude} &= g_{\rm syn}\tau_s\sigma\left(\frac{\tau_e}{\tau_D}\right)^2\frac{\tau_D}{\tau_D+\tau_e}\sqrt{\frac{1+\omega^2(\tau_D^2+\tau_e^2)}{1+\omega^2\tau_e^2}} \qquad (22) \\
\text{phase} &= \arctan\frac{\omega\tau_D}{1+\omega^2\tau_e(\tau_e+\tau_D)} \qquad (23)
\end{aligned}
$$

These quantities are very close to the amplitude and phase of the transfer function calculated from the synaptic response to a white noise input rate before (see Figure 3). In addition, the derivation of these quantities allows for the identification of the source of this behavior: The temporal modulation in the output is primarily determined by the sum of the mean rate times the synaptic efficacy modulation plus the mean synaptic efficacy times the presynaptic rate modulation. As these two temporal modulations are practically out of phase, they nearly cancel out each other and the small amplitude of the sum depends primarily on the phase

difference φ between the two, which grows for increasing stimulation fequency ω. This generates the fluctuation amplitude that grows with ω in the final result (Figure 3). The approximate differentiation performed by this network thus relies not only on the dynamics of its synapses, but also critically on the summation of asynchronous inputs at the postsynaptic site.
